# Do medical student mental stress and burnout vary with virtual versus in-person residency interviews

**DOI:** 10.2144/fsoa-2021-0046

**Published:** 2021-10-22

**Authors:** Dani Zoorob, Kara Richardson, Korina Gaishauser, Benjamin Hinkel, Hind N Moussa, James Van Hook, Rose A Maxwell

**Affiliations:** 1 Department of Obstetrics & Gynecology Academic Offices, University of Toledo, College of Medicine & Life Sciences, 2142 N. Cove Blvd., Toledo, OH 43606, USA; 2 Department of Obstetrics & Gynecology, University of Cincinnati College of Medicine, Kettering Maternal Fetal Medicine, Cincinnati, OH 45221, USA; 3 Department of Obstetrics & Gynecology, Wright State University, 128 E. Apple St., Weber CHE, Suite 3800 Dayton, OH 45409, USA

**Keywords:** applications, burnout, interview, medical student, NRMP, obstetrics and gynecology, residency, stress, virtual

## Abstract

**Aim:**

This study aimed to identify medical student stressors and mitigation methodologies based on interview modality.

**Materials & methods:**

A survey was administered to obstetrics and gynecology applicants in in-person (IP) and virtual (VR) National Resident Matching Program cycles. This included demographics, the Mayo Clinic Medical Students Well-Being Index and stressor questions.

**Results:**

A total of 137 of 151 surveys were completed (91% response rate). Subjective stress was significant in 76% of IP and 57% of VR applicants (p = 0.07). The objective Mayo Clinic Medical Students Well-Being Index values were higher in the IP (2.47 ± 1.75) compared with the VR group (2.00 ± 1.55; p = 0.10), suggesting lower stress with VR interviews. More IP (53%) compared with VR applicants (44%) were deemed ‘at risk’ (p < 0.01).

**Conclusion:**

VR interviews may mitigate select stressors during interviews.

## Lay abstract

Medical students encounter a significantly increasing amount of stress over the course of their training, with an acute rise noted when interviews occur near the conclusion of their undergraduate medical education. This study aimed to identify causes and potential mitigators of interview stressors based the modality of engagement, virtual or in person. Our survey results suggest that students subjectively felt and objectively encountered significant stress during both modalities; although, this was more pronounced during in-person interviews. Furthermore, the study suggests that virtual interviews may be the modality associated with less overall stress during the residency application cycles.

## Graphical abstract

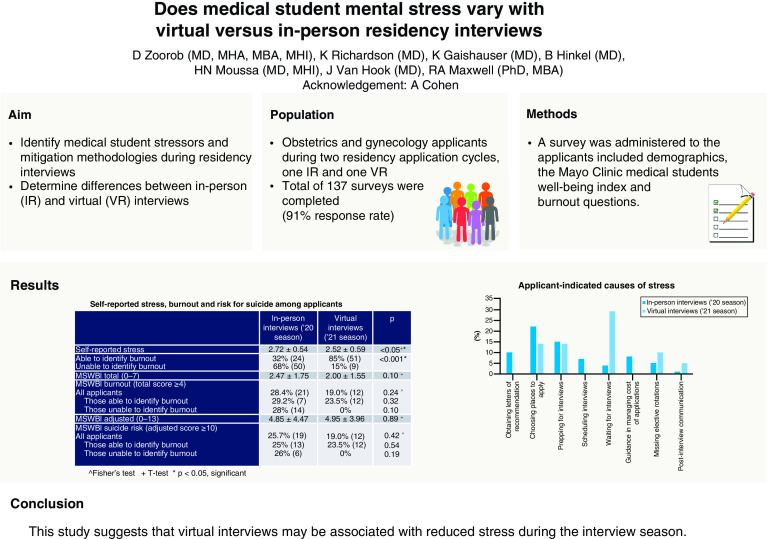

Medical student stress is a significant problem and has been shown to increase over the 4 years of training [1]. Medical student stress has been associated with decreased sleep quality [2]. Additionally, medical student stress has been shown to correlate with depression and anxiety [1,3]. Furthermore, medical student stress has been shown to be associated with decreased empathy for patients and increased burnout [1,4]. Given the significance of this issue, medical student stress and ultimately burnout, remain an important area of research.

Burnout has been well documented as a significant problem impacting physicians, residents and medical students throughout their careers [5–7]. While burnout has increasingly been addressed by studies and organizations including the American Medical Association, there has been limited research addressing medical student burnout rates specifically during the application cycle of the National Resident Matching Program (NRMP) [8]. In a 2011 study, it was found that 49% of responding 4th year medical students had at least one symptom of severe burnout, 38% endorsed symptoms of depression, 34% had low mental quality-of-life and around 14% had recent suicidal ideation [9]. A 2019 study found that the prevalence of emotional exhaustion, depersonalization and burnout peaked at the end of the 1st and 3rd years of medical school, with the highest rates being at the end of the 3rd year leading into the beginning of the application cycle [10]. That study suggested that burnout rates may vary during various time periods in medical school with high rates of burnout noted at the end of the 3rd year (59%) and a significant reduction by the end of the 4th year (38%). As this peak coincides with the recent transition of medical students into the clinical arena, the period when decisions for specialties are made, and the time when interview applications are concluded, the evidence suggests that the earlier part of the 4th year for medical students may be a highly stressful period of medical school resulting in augmented susceptibility to those already at risk of burnout.

As the COVID-19 pandemic impacted the globe, travel restrictions and infection concerns surfaced impacting the ability to host in-person (IP) interviews for residency applicants. Although, the change to online interviews for the 2020–2021 NRMP season has been associated with a lot of trepidation related to the transition to virtual (VR) interviews, direct comparison of variations between the two interview modalities on student stress has not been adequately reported to date.

The rationale for the study stems from the aforementioned peak in student stress and reported burnout that coincides with the period when a switch in interview modalities was made due to the pandemic. Thus, the primary aim of this study was to identify factors contributing to student perceived stress during the NRMP match season with focus on potential discrepancies resulting from the VR and IP interview approaches. Secondary aims included examination of the significance of stress on mental wellbeing, modalities that may help mitigate such concerns, as well as identifying burnout rates within the interview timeframes *per se*.

## Materials & methods

### Study design & participants

This was a cross sectional study of medical students applying to the OBGYN residency programs in both 2019–2020 and 2020–2021 applicant NRMP cycles. The former group had IP interviews conducted; whereas, the latter group interviewed through a VR platform.

The study was conducted at one site (the University of Toledo Obstetrics and Gynecology Residency Program) over 2 years and was approved by the ProMedica Health System institutional review board (IRB 19-081). An opt-out option was provided with anonymity maintained. Assistance for students for psychological support was offered, if desired by the interviewee.

### Data collection method

The study was conducted using a voluntary and anonymous survey. Informed consent was provided via a cover letter and was distributed prior to survey initiation with anonymity maintained at all times. Surveys for IP interview applicants were in the paper format with each being placed in a sealed envelope upon completion. VR interview applicants received a link to an online anonymous survey. The survey included the validated Mayo Clinic MedEd Web Solutions Well-Being Index for Medical Students (MSBWI) which helps identify increased risk of burnout with the total score and suicide risk with the adjusted score. Its score correlates with quality of life, fatigue, recent suicidal ideation, burnout, the likelihood of seriously considering dropping out of medical school and recent suicidal ideation. The survey also included closed-ended questions formulated to assess perceived causes of stress during the application cycle specifically, and potential solutions thereafter. Variation between the two surveys was minimal and reflected rewording necessary for VR versus IP stressors and opportunities offered. Open-ended queries captured suggestions for promoting a decrease in NRMP stress.

### Data analysis

Data were analyzed using SPSS (IBM SPSS Statistics for Windows, Version 27.0. IBM Corp, NY, USA). Categorical variables were compared using Chi-square and Fisher’s exact test. Continuous variables were compared using *t*-test. A p-value < 0.05 was considered significant. A one sample *t*-test was used to compare our sample to the national average for the Mayo Clinic Medical Students Well-Being Index (MSWBI). A z-test was used to compare the proportion of applicants with MSWBI≥4 from our sample with the national percentage.

## Results

A total of 151 surveys were distributed with 137 completed (91% cumulative response rate; IP: 74/82, VR: 63/69). The majority of respondents in both groups were between 25 and 27 years of age (IP: 80%; VR: 62%), reported being female (IP: 88%; VR: 81%) and were from the Midwest (IP: 87%; VR: 44%) (Table 1).

**Table 1.** Applicant demographics and details.

	IP interviews (2020 season) (n = 74)	VR interviews (2021 season) (n = 63)	p-value
Age <25 25–27 28–30 >30	5.4% (4) 79.7% (59) 12.2% (9) 2.8% (2)	0 61.9% (39) 28.6% (18) 9.5% (6)	<0.005^†^
Gender Female Male	87.8% (65) 12.2%	81.0% (51) 19.0% (12)	0.35
Region in the US Northeast South Midwest West	5.4% (4) 2.7% (2) 86.5% (64) 5.4% (4)	30.2% (19) 4.8% (3) 44.4% (28) 20.6% (13)	<0.001^†^
Number of applications felt necessary to be filed 21–30 31–40 41–50 51–60 61–70 71–80 >80	5.4% (4) 4.1% (3) 25.7% (19) 24.3% (18) 13.5% (10) 5.4% (4) 21.6% (16)	4.8% (3) 14.3% (9) 9.5% (6) 14.3% (9) 9.5% (6) 14.3% (9) 33.3% (21)	<0.02^†^

† p < 0.05, significant.

IP: In-person; VR: Virtual.

### Subjective stress assessment

Student perception of interview stress suggested higher rates for IP applicants than for VR applicants (IP = 2.72 ± 0.54; VR = 2.52 ± 0.59; p < 0.05), with 76% (n = 56) of the IP and 57% (n = 36) of VR applicants reporting extreme stress during the interview season (p = 0.07).

### Objective stress, burnout & suicidal likelihood assessment

Overall, IP applicants demonstrated higher medical student stress on the MSWBI total score and higher risk for suicide using the MSWBI adjusted score (Table 2). Both groups had lower MSWBI total scores than the national (NAT) average, but only the VR applicants scores were significantly lower than the national average (NAT = 2.59 ± 1.85 vs VR = 2.00 ± 1.55; p < 0.005). The VR applicants, but not the IP applicants, had a significantly lower rate of burnout (MSWBI≥4) compared with the national percentage (NAT = 31% vs VR = 19%; p < 0.05).

**Table 2.** Self-reported stress, burnout and risk for suicide among applicants.

	IP interviews (2020 season)	VR interviews (2021 season)	p-value
Self-reported stress	2.72 ± 0.54	2.52 ± 0.59	<0.05^‡^
Able to identify burnout Unable to identify burnout	32% (24) 68% (50)	85% (51) 15% (9)	<0.001^‡^
MSWBI total (0–7)	2.47 ± 1.75	2.00 ± 1.55	0.10
MSWBI burnout (total score ≥4) All applicants Those able to identify burnout Those unable to identify burnout	28.4% (21) 29.2% (7) 28% (14)	19.0% (12) 23.5% (12) 0%	0.24^†^ 0.32 0.10
MSWBI adjusted (0–13)	4.85 ± 4.47	4.95 ± 3.96	0.89
MSWBI suicide risk (adjusted score ≥10) All applicants Those able to identify burnout Those unable to identify burnout	25.7% (19) 25% (13) 26% (6)	19.0% (12) 23.5% (12) 0%	0.42^†^ 0.54 0.19

† Fisher’s test.

‡ p < 0.05, significant.

IP: In person; MSWBI: Mayo Clinic Medical Students Well-Being Index; VR: Virtual.

IP applicants were least likely to report being able to identify burnout (IP: 32% vs VR: 85%; p < 0.001). Among those not able to identify burnout (IP: 68% vs VR: 15%), only IP applicants were actually classified as burned out with MSWBI total scores ≥4 (IP: 28% vs VR: 0%; p < 0.10) and only IP applicants were actually classified as high risk for suicide by having a MSWBI adjusted score ≥10 (IP: 26% vs VR: 0%; p < 0.19). In addition, among those who reported that they were able to identify burnout, both IP and VR applicants could be classified as burned out (IP: 29.2% vs VR: 23.5%; p < 0.32) and at high risk for suicide (IP: 25% vs VR: 23.4%; p < 0.54).

### Stressor identification

The most prominent stressor for both IP and VR applicants was waiting for initiation of interviews, followed by choosing places to apply for the VR applicants and cost of applications for the IP applicants (Table 3). VR applicants were more likely to rate waiting for interviews as a contributor to burnout, while IP applicants were more likely to identify choosing places to apply, obtaining letters of recommendation and scheduling interviews as contributors to burnout (Table 4). Mutually exclusive stressors included cost of travel, followed by navigating new cities and then time lost on rotations during the trip for IP interviews; whereas, the VR meet and greets, followed by the interview locale and setup and then the cost of technology were the stressors for VR interviews.

**Table 3.** Stressor variations between interview modalities.

	IP interviews (2020 Season)	VR interviews (2021 Season)	p-value
Obtaining letters of recommendation	1.30 ± 0.92	1.76 ± 0.76	<0.002^†^
Choosing places to apply	1.70 ± 0.79	2.10 ± 0.76	<0.005^†^
Cost of applications	2.04 ± 1.01	1.76 ± 0.76	0.08
Cost to interview (traveling or technology)	2.01 ± 1.03	1.05 ± 0.96	<0.001^†^
Scheduling interviews	2.00 ± 0.84	1.86 ± 0.89	0.34
Waiting for interviews	2.45 ± 0.78	2.33 ± 0.84	0.42
Post-interview communication	1.43 ± 0.83	1.43 ± 1.01	0.99

Likert scale 0–4, where none = 0, extensive = 4.

† p < 0.05, significant.

IP: In person; VR: Virtual.

**Table 4.** Applicant-identified stressors contributing to burnout.

	IP interviews (2020 season)	VR interviews (2021 season)	p-value
Obtaining letters of recommendation	9.5% (7)	0	0.02
Choosing places to apply	21.6% (16)	14.3% (9)	0.38
Prepping for interviews	14.9% (11)	14.3% (9)	0.99
Scheduling interviews	6.8% (5)	0	0.07
Waiting for interviews	4.1% (3)	28.6% (18)	<0.001^‡^
Guidance in managing cost of applications	8.1% (6)	0	<0.04^‡^
Missing elective rotations/making up time on rotations	5.4% (4)	9.5% (6)	0.52
Post-interview communication	1.4% (1)	4.8% (3)	0.34

† Fisher’s exact test.

‡ p < 0.05, significant.

IP: In person; VR: Virtual.

### Medical school support

Medical schools were reported to check in on the applicants more often during the VR process compared with when travel was involved. Up to 32% (n = 24) of the IP and 19% (n = 12) of VR applicants reported that their home institutions did not check-in on them once interviewing started (Table 5). About 85% (n = 63) of IP applicants suggested that their home institutions could have supported them further in decreasing stress during the interview season while only 24% of VR applicants indicated so (p < 0.001). VR applicants reported feeling more prepared for interviews than IP applicants (IP = 2.47 ± 0.98 vs VR = 2.86 ± 0.71; p < 0.01).

**Table 5.** Rating of applicant-perceived institutional support.

	IP interviews (2020 season)	VR interviews (2021 season)	p-value
Submitting the application	2.15 ± 1.30	2.00 ± 1.21	0.50
Overview of what to expect at the interviews	2.12 ± 1.28	2.56 ± 1.28	0.06
Appearance at the interviews	2.05 ± 1.37	2.10 ± 1.46	0.87
Behavior/professionalism at the interviews	2.04 ± 1.55	2.43 ± 1.23	0.11
How to answer interview questions	2.07 ± 1.22	1.98 ± 1.14	0.69
Understanding the SOAP process	43.2% (32)	42.9% (27)	0.99
Feeling adequately prepared and ready for the interviews	2.47 ± 0.98	2.86 ± 0.71	<0.01^†^
Medical school informing students of a pre-existing check-in routine	79.7% (59)	28.6% (18)	<0.001^†^
Frequency of institutional check-in throughout the interview trail Never 1–2 times 3–4 times 5–6 times	32.4% (24) 52.7% (39) 10.8% (8) 4.1% (3)	19% (12) 47.6% (30) 28.6% (18) 4.8% (3)	<0.05^†^

Likert scale 0–4, where none = 0, extensive = 4.

† p < 0.05, significant.

IP: In person; SOAP: Supplemental Offer and Acceptance Program; VR: Virtual.

### Interview specific variables

Of the IP applicants, 49% had at least ten interview trips booked for the season. Up to 62% of VR applicants suggested that they would been ready to travel to at least ten interviews had the season converted to IP interviews at the last min. Additionally, VR applicants reported submitting more applications compared with the IP season (p < 0.02).

## Discussion

The purpose of this study was to compare the experiences of applicants participating in interviews during two NRMP match seasons that differed by interview modality (IP vs VR) and to identify potential causes of stress and burnout that 4th-year medical students face during this time. The two cohorts that were compared had not been offered the option of choice between formats, and as such there were no direct notable confounders that would suggest increased demonstration of commitment or suspected weakness in either route.

Our study shows that between 19% (VR) and 28% (IP) of medical students manifest signs of above-average stress during interview season. This translates to a nine-fold increase in the risk of burnout and a four-fold surge in an inferior quality of life [11,12]. Although prior studies have indicated transient increases in burnout as medical school progresses, our results specifically show high stress and burnout during the interview season [13]. We also noted a similar pattern (19% VR and 26% IP) for scores related to risk for suicide, which were well above previously documented rates of suicide ideation (11%) among medical students in general [2]. In addition, the VR applicants had higher scores for feeling adequately prepared and ready for interviews, which also may have helped to reduce their stress. These results suggest the need for focused attention toward applicants during the interview season, and potentially engaging them in directed stress management, mitigation education and heightened burnout and suicide awareness.

The study’s objective was also to identify resources that could assist with stress reduction. Both groups reported choosing which institutions to apply to as a significant contributor to stress, and this was more pronounced in the VR applicants. Although many online resources exist to help guide the students, multiple factors may contribute to the triggers thus limiting their extenuation such as grades, electives chosen, personal preferences and career aspirations. Applicants also indicated that their home institutions could help by explaining the interview process further, guiding them in managing travel costs, assistance with obtaining letters of recommendation and helping to prepare them better for the actual interviews. In addition, applicants identified other opportunities for support while on the interview trail in that 19–32% of applicants reported that their institution did not have a check-in during this time and 57% of applicants indicated that they did not understand the SOAP process. Many medical schools aggressively address preparation for interviews, but a standardized methodology for assistance or follow-up on the applicants while actively interviewing has not been described. IP applicants reported apprehension regarding health, safety and flight delays while traveling; whereas, VR interview trepidations centered around the VR ‘meet and greets’ and the format of the interviews. Panel interviews used during VR interviews were considered as more intimidating and did not offer the opportunity to connect as favorably with the interviewers. Although, the majority of IP applicants reported knowledge of pre-existing check-in routines, VR applicants were not as cognizant of such practices. This is likely due to the novelty of the online process, limited VR guidance published by undergraduate medical education authorities and the focus by medical schools on what they are more familiar with, in other words, preparation of interview portfolios including letters, application completion, mock interviews.

Our results concur with prior studies indicating significant financial stress resulting from the increased number of applications subjectively considered necessary as well as the travel costs incurred from IP interviews that ensue [14,15]. Although some may be mitigated by the change to VR format, others will persist. Similar numbers of applicants were ready to invest in applications to more than 50 programs in either interview season (IP: n = 48, 65%; VR: n = 45, 71%; p = 0.01). Although the minor percentage increase could be due to surplus fund availability to subsequent travel cancellation, the applicant portfolios and competitiveness could explain the increase in application numbers – but would not explain the larger geographic range of the applicants noted. This data shows that financial stress is a significant cause of stress for students with IP interviews, which is essentially eliminated with VR interviews.

With the change to a VR interview season in 2021, three factors reported by IP applicants became non issues: cost of travel, difficulty navigating cities and making up for significant travel time lost from rotations. However, these were replaced with new stressors including cost of upgrading the technology, getting to know the program and connecting with online interviewers. As the effects on stress and burnout in regard to the switch to VR interviews is currently lacking, it may be worth further assessing the newer parameters such as novel expenses of interviewing from home (equipment such as camera and computer), navigating nonstandardized interview software, as well as loss of the interpersonal interaction component of the interviews where body language and conversational cues play a role. Still, VR interviews were found to be significantly less stressful than IP interviews, suggesting that these new stressors may be inconsequential.

To date, few assessments targeting this critical time in medical students’ lives have been documented and potent interventions to decrease the incidence of applicant burnout seem scarce. Some studies have documented medical student burnout throughout medical school, but their focus has not included the time period covering interview season [6,13]. We believe that our study supplements the current literature and provides new information about the trade-offs associated with VR interviewing which seemed to eliminate some of the major stressors associated with the traditional IP interview season, such a time away from rotations, a shift generated by the pandemic that may be to the advantage of residency applicants.

Strengths of the study include the high response rate, the anonymous survey format, and the use of a validated medical student wellbeing index. Limitations of this study include being a voluntary survey that partially incorporated nonvalidated questions, restriction to only obstetrics and gynecology residency applicants and administration at only one institution reducing generalizability to other specialties and programs. However, the head-to-head comparison does help reduce such confounders and sheds light on the students needs which may not vary significantly among specialties. Knowing that the MSWBI has not been tested specifically for interview processes and only addresses the previous month during its assessment, another limitation relates to the respondents not being asked about recent major life events (such as the impact of COVID and other personal concerns) that could have contributed to their feelings of stress and burnout.

## Conclusion

Medical school students are markedly exposed to stress during training, and this gets particularly amplified during the residency applications and interview season. Whether these interviews are IP or VR, the stressors persist although VR modalities seem to be associated with reduced levels overall. Further studies are needed to compare interview modalities on a larger scale and across specialties.

## Future perspective

Studies are needed to identify stressors in both IP and VR interview modalities, improve medical student stress management during the interview season and provide information to institutions about opportunities to provide specific support as medical students enter this stressful period.

**Summary points**

- Limited comparative information is known about in-person (IP) and virtual (VR) interviewee psychological stressors during residency interviews.
- Subjective stress during interviews is perceived as significant in 76% of IP compared with 57% of VR applicants.
- Objective Mayo Clinic Medical Students Well-Being Index values were higher in IP compared with the VR applicants suggestive of reduced interview stress encountered in VR settings.
- More IP applicants (53%) compared with VR (44%) were deemed ‘at risk’.
- Up to 10% of ‘at-risk’ IP applicants were in the highest risk categories (Mayo Clinic Medical Students Well-Being Index 6 and 7) while none were in the VR group.
- The study suggests that IP interviews, compared with the VR option, may be associated with increased stress during the interview season.
- Among those who were not able to identify oneself as burned out in either group, only IP applicants were objectively classified as such (IP 28% vs VR 0%; p < 0.10).
